# On *k*th-Order Slant Weighted Toeplitz Operator

**DOI:** 10.1155/2013/960853

**Published:** 2013-07-17

**Authors:** S. C. Arora, Ritu Kathuria

**Affiliations:** ^1^Department of Mathematics, University of Delhi, Delhi 110007, India; ^2^Department of Mathematics, Motilal Nehru College, University of Delhi, Delhi 110007, India

## Abstract

Let *β* = {*β*_*n*_}_*n*∈*ℤ*_ be a sequence of positive numbers with *β*_0_ = 1, 0 < *β*_*n*_/*β*_*n*+1_ ≤ 1 when *n* ≥ 0 and 0 < *β*_*n*_/*β*_*n*−1_ ≤ 1 when *n* ≤ 0. A *k*th-order slant weighted Toeplitz operator on *L* ^2^(*β*) is given by *U*_*ϕ*_ = *W*_*k*_ *M*_*ϕ*_, where *M*_*ϕ*_ is the multiplication on *L* ^2^(*β*) and *W*_*k*_ is an operator on *L* ^2^(*β*) given by *W*_*k*_ *e*_*nk*_(*z*) = *(β*_*n*_/*β*_*nk*_)*e*_*n*_(*z*), {*e*_*n*_(*z*) = *z* ^*k*^/*β*_*k*_}_*k*∈*ℤ*_ being the orthonormal basis for *L* ^2^(*β*). In this paper, we define a *k*th-order slant weighted Toeplitz matrix and characterise *U*_*ϕ*_ in terms of this matrix. We further prove some properties of *U*_*ϕ*_ using this characterisation.

## 1. Introduction

Toeplitz operators and slant Toeplitz operators [1] have been found immensely useful, especially in the study of prediction theory [2], wavelet analysis [3], and solution of differential equations [4]. Originally, these operators were defined and studied on the usual *H* ^2^ and *L* ^2^ spaces. During the past few decades, different generalisations of these spaces, like the weighted function spaces *H*_*w*_^*P*^ and *L*_*w*_^*P*^ and the weighted sequence spaces *H* ^2^(*β*) and *L* ^2^(*β*) have developed. Shields [5] has made a systematic study of the multiplication operator on *L* ^2^(*β*). Lauric [6] has discussed the Toeplitz operator on *H* ^2^(*β*). Motivated by these studies, we introduced and studied the notion of a slant weighted Toeplitz operator [7] on *L* ^2^(*β*). In this paper, we study a *k*th-order slant weighted Toeplitz operator on the space *L* ^2^(*β*). We begin with the following preliminaries.

Let *β* = {*β*_*n*_}_*n*∈*ℤ*_ be a sequence of positive numbers with *β*_0_ = 1, 0 < (*β*_*n*_/*β*_*n*+1_) ≤ 1 when *n* ≥ 0 and 0 < (*β*_*n*_/*β*_*n*−1_) ≤ 1 when *n* ≤ 0.

Throughout the paper, we assume that for a fixed integer *k* ≥ 2, *β*_*k*_*n*__/*β*_*n*_ ≤ *M* < *∞*. Consider the following spaces [5, 6]:

(1) L2(β)={f(z)=∑n=−∞∞anzn | an∈ℂ,||f||β2=∑n=−∞∞|an|2βn2<∞},H2(β)={f(z)=∑n=0∞anzn | an∈ℂ,||f||β2=∑n=0∞|an|2βn2<∞}.

Then, (*L* ^2^(*β*), ||·||_*β*_) is a Hilbert space [6] with an orthonormal basis given by {*e*_*k*_(*z*) = *z* ^*k*^/*β*_*k*_}_*k*∈*ℤ*_ and with an inner product defined by

(2) $$\begin{array}{c} \langle \sum_{n=-\infty }^{\infty }a_{n}z^{n},\sum_{n=-\infty }b_{n}z^{n}\rangle =\sum_{n=-\infty }^{\infty }a_{n}{\overset{-}{b}}_{n}\beta_{n}^{2}. \end{array}$$

Also, *H* ^2^(*β*) is a subspace of *L* ^2^(*β*). Further, the space

(3) L∞(β)={ϕ(z)=∑n=−∞∞anzn | ϕL2(β)⊆L2(β),  ∃c∈R  such  that  ||ϕf||β≤c||f||β  ∀f∈L2(β)}

is a Banach space with respect to the norm defined by

(4) $$\begin{array}{c} {||\phi ||}_{\infty }=\inf\left\{c\;|{||\phi f||}_{\beta }\leq c{||f||}_{\beta }\;\forall f\in L^{2}\left(\beta \right)\right\}. \end{array}$$

The mapping *P* : *L* ^2^(*β*) → *H* ^2^(*β*) is the orthogonal projection of *L* ^2^(*β*) onto *H* ^2^(*β*). For a given *ϕ* ∈ *L* ^*∞*^(*β*), the induced weighted multiplication operator [5] is denoted by *M*_*ϕ*_ and is given by *M*_*ϕ*_ : *L* ^2^(*β*) → *L* ^2^(*β*) such that

(5) Mϕek(z)=1βk∑n=−∞∞anβn+ken+k(z)=1βk∑n=−∞∞an−kβnen(z).

If we put *ϕ*_1_(*z*) = *z*, then *M*_*ϕ*_1__ = *M*_*z*_ is the operator defined as *M*_*z*_ *e*_*k*_(*z*) = *w*_*k*_ *e*_*k*+1_(*z*), where *w*_*k*_ = *β*_*k*+1_/*β*_*k*_ for all *k* ∈ *ℤ*, and it is known as a weighted shift [5].

The slant weighted Toeplitz operator [7] *A*_*ϕ*_ is an operator on *L* ^2^(*β*) defined as *A*_*ϕ*_ : *L* ^2^(*β*) → *L* ^2^(*β*) such that *A*_*ϕ*_ *e*_*k*_(*z*) = (1/*β*_*k*_)∑_*n*=−*∞*_^*∞*^ *a*_2*n*−*k*_ *β*_*n*_ *e*_*n*_(*z*).

If *W* : *L* ^2^(*β*) → *L* ^2^(*β*) is such that

(6) $$\begin{array}{c} We_{2n}\left(z\right)=\frac{\beta_{n}}{\beta_{2n}}e_{n}\left(z\right),\;We_{2n-1}\left(z\right)=0\;\forall n\in \mathbb{Z}, \end{array}$$

then *A*_*ϕ*_ can be alternately defined by

(7) $$\begin{array}{c} A_{\phi }\left(f\right)=WM_{\phi }\left(f\right)=W\left(\phi f\right)\;\forall f\in L^{2}\left(\beta \right). \end{array}$$

## 2. *k*th-Order Slant Weighted Toeplitz Operator

Suppose that the operator *W*_*k*_ : *L* ^2^(*β*) → *L* ^2^(*β*) is such that

(8) $$\begin{array}{c} W_{k}e_{n}\left(z\right)=\{\begin{array}{cc} \frac{\beta_{n/k}}{\beta_{n}} & e_{n/k}\left(z\right)\;\text{if}\;n\;\text{is}\;\text{divisible}\;\text{by}\;k,\\ 0 & \text{otherwise}, \end{array} \end{array}$$

Then the matrix of *W*_*k*_ is

(9) $$\begin{array}{c} \left[\begin{array}{ccccccc} \cdots & \cdots \;\cdots \;\cdots \;\cdots & \cdots & \cdots & \cdots & \cdots & \cdots \\ \cdots & \frac{\beta_{0}}{\beta_{0}}\;\;0\;\;0\;\;0 & 0 & 0 & 0 & 0 & \cdots \\ \cdots & 0\;\;0\;\;0\;\;0 & \frac{\beta_{1}}{\beta_{k}} & 0 & \cdots & \cdots & \cdots \\ & \underset{k\text{-times}}{\underset{︸}{\;\;\;\;\;\;\;\;\;\;\;\;\;\;\;\;\;\;\;\;\;\;\;\;\;\;\;\;}} & & & & & \\ \cdots & 0\;\;0\;\;0\;\;0 & \cdots & \cdots & 0 & \frac{\beta_{2}}{\beta_{2k}} & \cdots \\ \cdots & \;0\;\;0\;\;\cdots \;\cdots & \cdots & \cdots & \cdots & \cdots & \cdots \\ \cdots & \cdots \;\cdots \;\cdots \;\cdots & \cdots & \cdots & \cdots & \cdots & \cdots \end{array}\right]. \end{array}$$

The adjoint of *W*_*k*_ is given by

(10) $$\begin{array}{c} W_{k}^{*}e_{n}\left(z\right)=\frac{\beta_{n}}{\beta_{kn}}e_{kn}\left(z\right)\;\forall n\in \mathbb{Z}. \end{array}$$

**Definition 1 (see [8])**

For an integer *k* ≥ 2, the *k*th order slant weighted Toeplitz operator *U*_*ϕ*_ : *L* ^2^(*β*) → *L* ^2^(*β*) is such that *U*_*ϕ*_(*f*) = *W*_*k*_ *M*_*ϕ*_(*f*) for all *f* ∈ *L* ^2^(*β*). Thus, *U*_*ϕ*_ *e*_*j*_(*z*) = (1/*β*_*j*_)∑_*n*=−*∞*_^*∞*^ *a*_*nk*−*j*_ *β*_*n*_ *e*_*n*_(*z*).

The (*i*, *j*)th entry of the matrix of *U*_*ϕ*_ is given by

(11) 〈Uϕej,ei〉=〈1βj ∑n=−∞∞ank−jβnen(z),ei(z)〉=aik−jβiβj.

Hence, the matrix of *U*_*ϕ*_ with respect to this basis is as follows:

(12) $$\begin{array}{c} \left[\begin{array}{cccccc} \cdots & \cdots & \cdots & \cdots & \cdots & \cdots \\ \cdots & a_{-k+1}\frac{\beta_{-1}}{\beta_{-1}} & a_{-k}\frac{\beta_{1}}{\beta_{0}} & a_{-k-1}\frac{\beta_{-1}}{\beta_{1}} & a_{-k-2}\frac{\beta_{-1}}{\beta_{2}} & \cdots \\ \cdots & a_{1}\frac{\beta_{0}}{\beta_{-1}} & a_{0}\frac{\beta_{0}}{\beta_{0}} & a_{-1}\frac{\beta_{0}}{\beta_{1}} & a_{-2}\frac{\beta_{0}}{\beta_{2}} & \cdots \\ \cdots & a_{k+1}\frac{\beta_{1}}{\beta_{-1}} & a_{k}\frac{\beta_{1}}{\beta_{0}} & a_{k-1}\frac{\beta_{1}}{\beta_{1}} & a_{k-2}\frac{\beta_{1}}{\beta_{2}} & \cdots \\ \cdots & a_{2k+1}\frac{\beta_{2}}{\beta_{-1}} & a_{2k}\frac{\beta_{2}}{\beta_{0}} & a_{2k-1}\frac{\beta_{2}}{\beta_{1}} & a_{2k-2}\frac{\beta_{2}}{\beta_{2}} & \cdots \\ \cdots & \cdots & \cdots & \cdots & \cdots & \cdots \end{array}\right]. \end{array}$$

**Theorem 2**

The mapping *ϕ* → *U*_*ϕ*_ is linear and one to one.

**Proof**

For linearity, consider

(13) Uαϕ+βψ=WkMαϕ+βψ=Wk(Mαϕ+Mβψ)=WkMαϕ+WkMβψ=αWkMϕ+βWkMψ=αUϕ+βUψ.

For checking one-to-one, let *U*_*ϕ*_ = *U*_*ψ*_.

Then, *U*_*ϕ*_ − *U*_*ψ*_ = 0. By linearity, we get *U*_*ϕ*−*ψ*_ = 0.

Hence,

(14) Uϕ−ψen(z)=0, n∈ℤ,WkMϕ−ψen(z)=0, n∈ℤ,Wk(ϕ−ψ)en(z)=0, n∈ℤ.

This implies that either *ϕ* − *ψ* = 0 or the degree of (*ϕ* − *ψ*)*e*_*n*_(*z*) is not divisible by *k*. But since this is true for all *n* ∈ *ℤ*, the second possibility is ruled out. Hence, *ϕ* − *ψ* = 0 or *ϕ* = *ψ*.

**Theorem 3**

Consider the following: (i) *M*_*z*_ *W*_*k*_ = *W*_*k*_ *M*_*z*^*k*^_; (ii) *M*_*z*^*m*^_ *W*_*k*_ = *W*_*k*_ *M*_*z*^*km*^_, *m* ∈ *ℤ*.

**Proof**

(i) It is sufficient to prove that

(15) $$\begin{array}{c} M_{z}W_{k}e_{n}\left(z\right)=W_{k}M_{z^{k}}e_{n}\left(z\right)\;\forall n\in \mathbb{Z}. \end{array}$$

Suppose that *n* is not a multiple of *k*. Then,

(16) $$\begin{array}{c} M_{z}W_{k}e_{n}\left(z\right)=M_{z}0=0=W_{k}M_{z^{k}}e_{n}\left(z\right). \end{array}$$

Now, when *n* = *pk* (multiple of *k*),

(17) MzWken(z)=Mzβpβpkep(z)=βp+1βpkep+1(z).

On the other hand,

(18) WkMzken(z)=WkMzkepk(z)=Wkβ(p+1)kβpke(p+1)k(z)=βp+1βpkep+1(z).

Hence from (17) and (18), we get that

(19) $$\begin{array}{c} M_{z}W_{k}e_{n}\left(z\right)=W_{k}M_{z^{k}}e_{n}\left(z\right)\;\text{whenever}\;n\;\text{is}\;\text{a}\;\text{multiple}\;\text{of}\;k. \end{array}$$

We therefore conclude that for all *n* ∈ *ℤ*,

(20) MzWken(z)=WkMzken(z)MzWk=WkMzk.

(ii) We prove the result by induction on *m*.

For *m* = 1, the result is true by part (i).

Suppose that the result is true for *m* = *l*. Then,

(21) $$\begin{array}{c} M_{z^{l}}W_{k}=W_{k}M_{z^{lk}}. \end{array}$$

Now

(22) Mz(l+1)kWk=MzMzlWk=MzWkMzlk=WkMzkMzlk=WkMz(l+1)k.

Hence by induction, the result is true for all *m* ∈ *Z* ^+^.

For *m* = −1 and 1 ≤ *j* ≤ *k* − 1,

(23) MzmWkenk+j(z)=Mz−1  0=0=WkMz−kenk+j(z).

For *m* = −1 and *j* = *nk* (a multiple of *k*),

(24) MzmWkej(z)=Mz−1Wkenk(z)=Mz−1βnβnken(z)=βnβnkMz−1en(z)=βnβnkβn−1βnen−1(z)=βn−1βnken−1(z).

On the other hand,

(25) WkMz−1enk(z)=Wk(z−1znkβnk)=Wkz(n−1)kβnkβ(n−1)kβ(n−1)k=β(n−1)kβnkWke(n−1)k(z)=β(n−1)kβnkβn−1β(n−1)ken−1(z)=βn−1βnken−1(z).

From (23), (24), and (25), we conclude that

(26) $$\begin{array}{c} M_{z^{-1}}W_{k}=W_{k}M_{z^{-k}}. \end{array}$$

Hence, the result is true for *m* = −1 also. Therefore, by using induction, we can prove it for all negative integers *m*. The case when *m* = 0 is trivially true.

Hence the theorem is true.

**Corollary 4**

Let *ϕ*(*z*) = ∑_*n*=−*∞*_^*∞*^ *a*_*n*_ *z* ^*n*^ ∈ *L* ^*∞*^(*β*). Then,

(27) $$\begin{array}{c} M_{\phi (z)}W_{k}=W_{k}M_{\phi (z^{k})}. \end{array}$$

**Theorem 5**

Consider the following: *M*_*ϕ*(*z*)_ *U*_*ψ*(*z*)_ = *U*_*ϕ*(*z*^*k*^)*ψ*(*z*)_.

**Proof**

The proof is as follows:

(28) Mϕ(z)Uψ(z)=Mϕ(z)WkMψ(z)=WkMϕ(zk)Mψ(z)=WkMϕ(zk)ψ(z)=Uϕ(zk)ψ(z).

## 3. Matrix Characterisation of *U*_*ϕ*_

**Definition 6**

Let *w*_*n*_ = *β*_*n*+1_/*β*_*n*_ for all *n* ∈ *ℤ*. Then, the *k*th order slant weighted Toeplitz matrix corresponding to the weight sequence 〈*w*_*n*_〉 is a bilaterally infinite matrix 〈*λ*_*ij*_〉 such that

(29) $$\begin{array}{c} \lambda_{i+1,j+k}=\frac{w_{i}}{w_{j}w_{j+1}\cdots w_{j+k-1}}\lambda_{i,j},\;i,j=0,\pm 1,\pm 2,\ldots . \end{array}$$

We have proved earlier [8] that *U* is a *k*th order slant weighted Toeplitz operator if and only if *M*_*z*_ *U* = *UM*_*z*^*k*^_.

Next, we prove a characterisation of the *k*th order slant weighted Toeplitz operator in terms of the matrix previously defined.

**Theorem 7**

A necessary and sufficient condition that an operator *U* on *L* ^2^(*β*) is a *k* th order slant weighted Toeplitz operator is that its matrix with respect to the orthonormal basis {*e*_*k*_(*z*) = *z* ^*k*^/*β*_*k*_}_*k*∈*ℤ*_ is a *k* th order slant weighted Toeplitz matrix.

**Proof**

Suppose that *U* is a *k*th order slant weighted Toeplitz operator. Then, the corresponding matrix 〈*λ*_*ij*_〉 is given by

(30) λij=〈Uej,ei〉=aki−jβiβj.

Further,

(31) λi+1,j+k=〈Uej+k,ei+1〉=aki+k−j−kβi+1βj+k=aki−jβi+1βj+k=βi+1βj+kβjβiλi,j=wiwjwj+1⋯wj+k−1λi,j,

where *w*_*n*_ = *β*_*n*+1_/*β*_*n*_ for all *n* ∈ *ℤ*. Thus, the matrix of  *U* is a *k*th order slant weighted Toeplitz matrix. Conversely, we assume that *U* is an operator on *L* ^2^(*β*) whose matrix is a *k*th order slant weighted Toeplitz matrix. This means that for all *i*, *j* ∈ *ℤ*, we have

(32) $$\begin{array}{c} \langle Ue_{j},e_{i}\rangle =\frac{w_{j}w_{j+1}\cdots w_{j+k-1}}{w_{i}}\langle Ue_{j+k},e_{i+1}\rangle . \end{array}$$

Now,

(33) 〈MzUej,ei〉=〈Uej,Mz∗ei〉=〈Uej,wi−1ei−1〉=wi−1〈Uej,ei−1〉=wi−1wjwj+1⋯wj+k−1wi−1×〈Uej+k,ei〉 (using  (32))=〈UMzkej,ei〉, ∀i,j∈ℤ.

Hence *M*_*z*_ *U* = *UM*_*z*^*k*^_. Therefore, we conclude that *U* is a *k*th order slant weighted Toeplitz operator.

Next, consider the operator *S* : *L* ^2^(*β*) → *L* ^2^(*β*) given by *Se*_*j*_ = (1/*w*_*j*_)*e*_*j*+1_. Then, S*e_*j*_ = (1/*w*_*j*−1_)*e*_*j*−1_.

Now, *S* is bounded as 〈*w*_*n*_〉 is always positive and bounded.

**Lemma 8**

Consider the following: S* = *M*_*z*_^−1^.

**Proof**

The proof is as follows:

(34) $$\begin{array}{c} S^{*}M_{z}e_{j}=S^{*}w_{j}e_{j+1}=\frac{w_{j}}{w_{j}}e_{j}=e_{j},\\ M_{z}S^{*}e_{j}=M_{z}\frac{1}{w_{j-1}}e_{j-1}=\frac{w_{j-1}}{w_{j-1}}e_{j}=e_{j}. \end{array}$$

**Theorem 9**

A bounded operator *U* on *L* ^2^(*β*) is a *k* th order slant weighted Toeplitz operator if and only if  *U* = *M*_*z*_^−1^ *UM*_*z*^*k*^_, where *M*_*z*_ and *M*_*z*^*k*^_, are the multiplication operators induced by *z* and *z* ^*k*^, respectively.

**Proof**

Let *U* be a *k*th order slant weighted Toeplitz operator on *L* ^2^(*β*). Then, from (32) we get that

(35) 〈Uej,ei〉=wjwj+1⋯wj+k−1wi〈Uej+k,ei+1〉=〈UMzkej,Sei〉=〈S∗UMzkej,ei〉=〈Mz−1UMzkej,ei〉, i,j=0,±1,±2,….

Thus, *U* = *M*_*z*_^−1^ *UM*_*z*^*k*^_.

For the converse part, we assume that *U* is a bounded operator on *L* ^2^(*β*) satisfying *U* = *M*_*z*_^−1^ *UM*_*z*^*k*^_ for some fixed integer *k* ≥ 2. Then, for all *i*, *j* ∈ *ℤ*,

(36) 〈Uej,ei〉=〈Mz−1UMzkej,ei〉=〈S∗UMzkej,ei〉=〈UMzkej,Sei〉wjwj+1⋯wj+k−1wi〈Uej+k,ei+1〉.

The previous equation shows that the matrix corresponding to *U* is a *k*th order slant weighted Toeplitz matrix. Hence, by Theroem 7, *U* is a *k*th order slant weighted Toeplitz operator.

**Corollary 10**

For a fixed integer *k* ≥ 2, the set of all *k* th order slant weighted Toeplitz operators is weakly closed and hence strongly closed.

**Proof**

We assume that for each positive integer *n*, *U*_*n*_ is a *k*th order slant weighted Toeplitz operator and *U*_*n*_ → *U* weakly. Then, for *f*, *g* ∈ *L* ^2^(*β*), we get that 〈*U*_*n*_ *f*, *g*〉→〈*Uf*, *g*〉.

From the previous theorem, this implies that

(37) 〈Mz−1UnMzkf,g〉=〈S∗UnMzkf,g〉=〈UnMzkf,Sg〉=〈Unzk·f,Sg〉→〈Uzk·f,Sg〉=〈Mz−1UMzkf,g〉.

But, for each *n*, *U*_*n*_ = *M*_*z*_^−1^ *U*_*n*_ *M*_*z*^*k*^_.

Hence, *U* = *M*_*z*_^−1^ *UM*_*z*^*k*^_. Therefore, *U* is a *k*th order slant weighted Toeplitz operator.

## 4. Compression to *H* ^2^(*β*)

**Definition 11**

The compression of a *k*th order slant weighted Toeplitz operator on *H* ^2^(*β*) is denoted by *V*_*ϕ*_ and defined as *V*_*ϕ*_ *e*_*j*_(*z*) = (1/*β*_*j*_)∑_*n*=0_^*∞*^ *a*_*nk*−*j*_ *β*_*n*_ *e*_*n*_(*z*).  *j* = 0,1, 2,….

Alternatively,

(38) Vϕ=PUϕ|H2(β)=PWkMϕ|H2(β)=WkPMϕ|H2(β) (since  P  reduces  Wk [8])=WkTϕ,

where *T*_*ϕ*_ : *H* ^2^(*β*) → *H* ^2^(*β*) is the Toeplitz operator on *H* ^2^(*β*) induced by *ϕ* ∈ *L* ^*∞*^(*β*). The matrix of *V*_*ϕ*_ is unilaterally infinite and has the form

(39) $$\begin{array}{c} \left[\begin{array}{ccccc} a_{0}\frac{\beta_{0}}{\beta_{0}} & & a_{-1}\frac{\beta_{0}}{\beta_{1}} & a_{-2}\frac{\beta_{0}}{\beta_{2}} & \cdots \\ a_{k}\frac{\beta_{1}}{\beta_{0}} & & a_{k-1}\frac{\beta_{1}}{\beta_{1}} & a_{k-2}\frac{\beta_{1}}{\beta_{2}} & \cdots \\ a_{2k}\frac{\beta_{2}}{\beta_{0}} & & a_{2k-1}\frac{\beta_{2}}{\beta_{1}} & a_{2k-2}\frac{\beta_{2}}{\beta_{2}} & \cdots \\ \cdots & & \cdots & \cdots & \cdots \end{array}\right] \end{array}$$

for a given *ϕ* = ∑_*n*=−*∞*_^*∞*^ *a*_*n*_ *z* ^*n*^.

Further, we observe the following.

- The mapping *ϕ* → *V*_*ϕ*_ is linear and one to one.
- *T*_*ϕ*_ *W*_*k*_ = *PM*_*ϕ*_ *W*_*k*_ = *PW*_*k*_ *M*_*ϕ*_(*z* ^*k*^) = *PU*_*ϕ*(*z*^*k*^)_ = *V*_*ϕ*_(*z* ^*k*^).
